# Fusion of Radiomics and Gated Graph Attention Network for Pulmonary Nodule Malignancy Classification

**DOI:** 10.3390/jimaging12090408

**Published:** 2026-08-30

**Authors:** Xinying Guo, Zirong Yu, Jibin Yin

**Affiliations:** Faculty of Information Engineering and Automation, Kunming University of Science and Technology, Kunming 650500, China; 20242104107@stu.kust.edu.cn (X.G.);

**Keywords:** pulmonary nodule, radiomics, graph attention network, multimodal feature fusion, malignancy classification

## Abstract

Pulmonary nodule malignancy classification requires effective integration of heterogeneous imaging features and contextual information among nodules. Existing models mainly analyze nodules independently and may overlook inter-nodule relationships. We propose RGGA-Net, a radiomics-guided graph attention network that integrates deep imaging features and radiomics representations through cross-modal interaction and graph-based reasoning. A learned edge gate is introduced to adaptively modulate graph message passing, and an anchor regularization strategy is used to improve representation stability. RGGA-Net was evaluated on the LUNA25 dataset using patient-level splitting with an internal held-out test set and further assessed on the LIDC-IDRI cohort under cross-dataset evaluation. On the internal test set, RGGA-Net achieved an AUC of 0.8910 and a PR-AUC of 0.5173. External evaluation on LIDC-IDRI demonstrated moderate discrimination (AUC = 0.7023). Gate–Anchor ablation analysis showed a favorable interaction pattern between the learned gate and anchor regularization, although statistical superiority was not established. These findings suggest that radiomics-guided graph attention provides a feasible framework for incorporating inter-nodule information into malignancy classification, while further multi-center validation remains necessary.

## 1. Introduction

The widespread adoption of low-dose computed tomography (LDCT) screening has substantially increased the incidental detection of pulmonary nodules [1,2,3]. However, the central clinical challenge in nodule management is not detection per se, but risk stratification: determining whether a detected nodule warrants active surveillance, further diagnostic workup, or intervention. This decision is inherently complex, involving the integration of heterogeneous imaging characteristics, including nodule density, morphology, margin features, internal texture, and contextual information from multiple nodules within the same CT examination [4,5].

In clinical practice, pulmonary nodules are primarily evaluated according to their individual imaging characteristics, including size, density, morphology, margin features, and internal texture. When multiple nodules are present within the same CT examination, comparisons among lesions may provide additional contextual information by revealing shared or distinct phenotypic patterns. Such inter-nodule relationships therefore represent a complementary source of information rather than a replacement for individual nodule assessment. This form of contextual information remains insufficiently explored in current computational models [4].

Computer-aided diagnosis systems for pulmonary nodule analysis have made considerable progress, yet an important gap remains between computational feature learning and clinical decision-making. Single-nodule deep learning models, including three-dimensional convolutional neural networks [6] and transformer-based architectures [7], achieve strong discriminative performance but mainly focus on individual nodule appearance and may overlook inter-nodule contextual information. Hybrid approaches combining deep features with handcrafted radiomics have demonstrated advantages over single-modality methods [8,9], but most approaches rely on direct feature concatenation and do not explicitly model cross-modal interactions within a shared representation space. Graph-based methods provide a natural framework for representing relationships among nodules; however, existing approaches often construct graphs based on spatial proximity or standard graph attention mechanisms [10,11], without incorporating radiomics-derived information into adaptive graph reasoning.

Three specific limitations therefore remain unresolved in the existing literature. First, multimodal fusion strategies based on direct concatenation may not fully exploit the complementary relationship between deep imaging features and quantitative radiomics descriptors [9,12]. Second, contextual relationships among multiple nodules within the same CT examination are rarely incorporated into malignancy classification models. Third, existing graph-based approaches have not fully explored how radiomics-derived phenotypic information can be used to guide adaptive information propagation between nodules. Recent benchmark studies, including the LUNA25 challenge, have further highlighted the importance of standardized external evaluation for AI-based lung nodule malignancy risk estimation and demonstrated the potential of AI systems under clinically relevant evaluation settings [13].

To address these limitations, we propose RGGA-Net, a radiomics-guided graph attention network for pulmonary nodule malignancy classification. RGGA-Net integrates three-dimensional deep features and handcrafted radiomics features through a cross-modal cross-attention fusion module, enabling complementary information exchange between different feature domains. A nodule-level graph is constructed using k-nearest-neighbor search in the radiomics feature space, allowing radiomics-derived characteristics to define inter-nodule relationships [10,11]. Within the graph attention layers, a learned radiomics-informed gate adaptively modulates edge contributions through attention recalibration, rather than relying on a predefined similarity function [14]. This design enables the model to incorporate phenotypic context during graph message passing while maintaining data-driven flexibility. The model is jointly optimized with focal loss for class imbalance and an anchor loss that encourages consistency between graph-learned embeddings and a radiomics-guided reference space.

In summary, this study makes the following contributions:

1. We propose RGGA-Net, a multimodal malignancy classification framework that combines cross-modal cross-attention fusion with radiomics-guided graph attention to incorporate inter-nodule contextual information.
2. We introduce a learned radiomics-informed gating mechanism that adaptively regulates graph message passing through attention modulation, incorporating radiomics-derived relationships into graph reasoning.
3. We comprehensively evaluate RGGA-Net using patient-level internal evaluation on the LUNA25 cohort and cross-dataset external evaluation on the LIDC-IDRI cohort, including ablation analysis, calibration assessment, decision-curve analysis, and cross-dataset external evaluation to investigate model performance and generalizability.

## 2. Materials and Methods

### 2.1. Datasets and Data Splitting

This study used the LUNA25 dataset for model development and internal held-out evaluation and the LIDC-IDRI dataset for cross-dataset external evaluation. Because the two datasets differ in both image acquisition characteristics and malignancy reference standards, the LIDC-IDRI experiment was interpreted as a cross-dataset external evaluation under imaging-domain and reference-standard shifts rather than as external clinical validation under an equivalent reference standard.

#### 2.1.1. LUNA25 Dataset

The original LUNA25 collection contained 6163 annotated nodules from 4069 CT scans of 2120 patients [13]. We excluded records for which a valid segmentation mask was unavailable or could not be reliably matched to the corresponding annotated nodule. After this quality-control procedure, the final analytic cohort comprised 6047 nodules from 4017 CT scans of 2096 patients, including 539 malignant and 5508 benign nodules. Importantly, the CT-based nodule annotations should be distinguished from the malignancy reference standard. In LUNA25, the malignancy endpoint was outcome-based: malignant nodules were confirmed through histopathological examination, whereas benign nodules were verified by longitudinal stability. For the public NLST development cohort used in this study, the LUNA25 protocol specifies a median benign follow-up period of at least six years [13].

To prevent patient-level information leakage, the dataset was partitioned with the patient as the grouping unit. All CT scans and nodules belonging to the same patient were assigned exclusively to the training, validation, or test subset. After enforcing the patient-level grouping constraint, stratified partitioning based on patient-level malignancy information was used to maintain the malignant and benign distributions as consistently as possible across the three subsets. Class distribution balance was assessed at the nodule level after partitioning, while the patient-level grouping constraint was strictly preserved throughout the splitting procedure. The resulting training set contained 1469 patients, 2844 CT scans, and 4289 nodules, including 362 malignant and 3927 benign nodules; the validation set contained 315 patients, 590 CT scans, and 888 nodules, including 83 malignant and 805 benign nodules; and the internal held-out test set contained 312 patients, 583 CT scans, and 870 nodules, including 94 malignant and 776 benign nodules. No patient overlap was observed between any pair of subsets.

To obtain three-dimensional nodule segmentation masks, we further used the LUNA25-MedSAM2 annotations [15]. These annotations were generated on the original LUNA25 CT scans using the MedSAM2 Lesion CT segmentation model with point prompts, followed by manual inspection and necessary correction [16]. The resulting masks were used for nodule ROI extraction, radiomics feature calculation, and model input construction.

Because the number of nodules within the same CT scan was associated with the prevalence of malignant nodules, we additionally report the distribution of single-nodule scans (N=1), two-nodule scans (N=2), and scans containing three or more nodules (N≥3). Here, *N* denotes the number of annotated nodules within the same CT scan. The detailed distributions are summarized in Table 1.

#### 2.1.2. LIDC-IDRI Dataset

The Lung Image Database Consortium and Image Database Resource Initiative (LIDC-IDRI) dataset was used for cross-dataset external evaluation [17]. The database contains 1018 thoracic CT cases annotated through a two-phase reading process involving up to four experienced thoracic radiologists. For each detected nodule, the available reader annotations include contour information and a malignancy-suspicion score ranging from 1 to 5. These scores represent radiological assessments rather than pathology-confirmed diagnostic labels.

For the primary external analysis, annotations belonging to the same nodule were grouped across readers, and a multi-reader consensus mask was generated at a consensus level of 0.5. Only nodules assessed by at least three radiologists were assigned an evaluation label. A nodule was categorized as benign when the median malignancy score was no greater than 2 and as malignant when the median score was no less than 4. Nodules with an intermediate median score were excluded from metric calculation but were retained as graph context nodes when their image patch, consensus mask, and radiomics features passed quality control.

LIDC-IDRI was used exclusively for external inference. No LIDC-IDRI samples were used for model training, model selection, hyperparameter optimization, radiomics standardization, temperature scaling, or classification-threshold selection. The image preprocessing settings, radiomics feature schema, training-set normalization parameters, graph construction parameters, and model weights were transferred directly from the LUNA25 development pipeline. Predictions from the three models trained with random seeds 2026, 2027, and 2028 were averaged at the probability level. Potential overlap between LUNA25 and LIDC-IDRI was assessed at the CT-series level using SeriesInstanceUID matching, canonical HU-volume hashing, and candidate image similarity checks, and any confirmed or suspected duplicate series were excluded from the external cohort. After preprocessing, cross-dataset deduplication, and quality control, the final external cohort comprised 875 patients, 883 CT scans, and 616 evaluable nodules, including 315 benign and 301 malignant nodules.

### 2.2. Overall Framework of RGGA-Net

**Figure 1:**
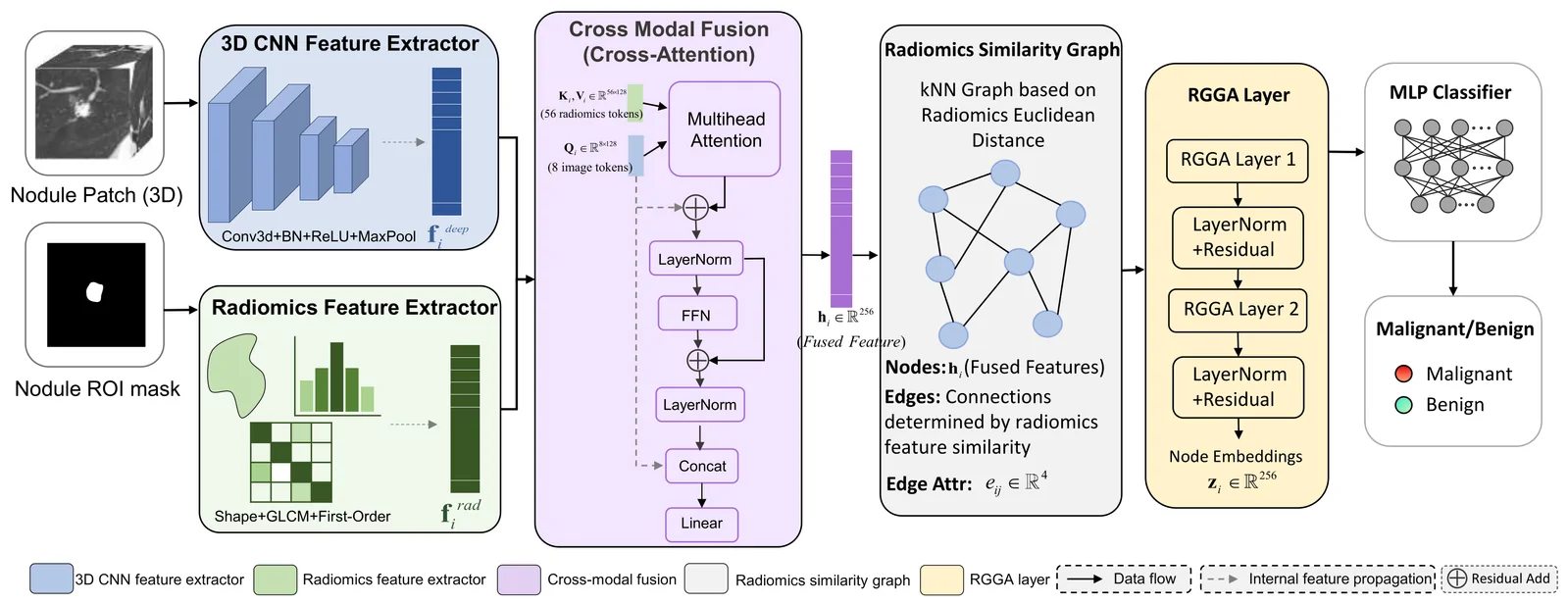
Overall framework of RGGA-Net. For each nodule, a 3D CNN and a radiomics feature extractor process the nodule patch and ROI mask in parallel, producing deep imaging features and a 56-dimensional handcrafted radiomics feature vector $f_{i}^{\mathrm{rad}}$. For cross-modal fusion, the final CNN feature map is converted into eight spatial image tokens, while the 56 standardized radiomics features are individually mapped into 56 radiomics tokens. Multi-head cross-modal attention uses the image tokens as queries and the radiomics tokens as keys and values, yielding a 256-dimensional fused node representation $h_{i}$. A radiomics similarity graph is then constructed by k-nearest-neighbor search in the standardized radiomics feature space, with 4-dimensional edge attributes $e_{ij}\in R^{4}$ encoding radiomics-derived inter-nodule differences. Two stacked RGGA layers with residual connections produce the final node embedding $z_{i}$, which is fed into an MLP classifier for malignancy prediction.

The overall framework of the proposed RGGA-Net is illustrated in Figure 1. For each pulmonary nodule, deep imaging features and handcrafted radiomics features were first extracted and integrated to obtain a multimodal nodule representation. Scan-level graphs were then constructed from multiple nodules, and the proposed radiomics-gated graph attention module was used to model inter-nodule relationships before node-level malignancy prediction.

### 2.3. CT Preprocessing and Nodule Patch Extraction

All CT scans were converted to Hounsfield units and clipped to the lung-window range of [−1000,400] HU. The clipped intensities were then linearly normalized to [0,1] for use as the input to the three-dimensional convolutional neural network.

For each CT scan, individual nodule instances were identified from the binary ROI mask using three-dimensional connected-component analysis. For each connected component, the voxel-space centroid $\left(z_{m},y_{m},x_{m}\right)$ was calculated and associated with the corresponding nodule label.

A fixed-size three-dimensional patch of 64×128×128 voxels in the depth, height, and width dimensions, respectively, was extracted around each nodule centroid. The patch was centered on $\left(z_{m},y_{m},x_{m}\right)$ to retain both the nodule and its surrounding perinodular context. When the cropping region extended beyond the CT volume boundary, the missing image region was padded using the lower clipping value of −1000 HU, while the corresponding mask region was padded with zeros. The cropped CT patch and its spatially aligned binary ROI mask were subsequently used for deep-feature and radiomics-feature extraction, respectively.

### 2.4. Deep Feature and Radiomics Feature Extraction

For each nodule, the preprocessed CT patch was processed by a lightweight three-dimensional convolutional neural network to extract deep semantic features. The feature extractor consisted of four sequential convolutional blocks, each comprising a 3D convolutional layer, batch normalization, a ReLU activation function, and 3D max pooling. The complete channel configuration, kernel size, stride, padding, and pooling settings are provided in Appendix A. The final convolutional feature map,

(1) $$F_{i}\in R^{C\times D\times H\times W},$$

was retained for the spatial-token construction described in Section 2.5. In parallel, global average pooling and a linear projection layer were applied to obtain a 256-dimensional global deep feature vector,

(2) $$f_{i}^{\mathrm{deep}}\in R^{256}.$$

Handcrafted radiomics features were extracted from the three-dimensional nodule ROI using PyRadiomics (version 3.1.0) following the feature definitions implemented in PyRadiomics and the Image Biomarker Standardisation Initiative nomenclature [18,19]. Radiomics extraction was performed on the CT images represented in Hounsfield units, rather than on the intensity-normalized tensors used as network inputs. Before feature extraction, CT intensities were clipped to the lung window of [−1000,400] HU. The CT images and corresponding binary masks had been resampled to an isotropic voxel spacing of $1\times 1\times 1\;{\mathrm{mm}}^{3}$ using B-spline interpolation for the images and nearest-neighbor interpolation for the masks. No additional resampling was performed within PyRadiomics.

Only the Original image type was enabled. Wavelet, Laplacian-of-Gaussian, and other filtered image types were not used. Gray-level discretization was performed using a fixed bin width of 25 HU. Three feature classes were enabled: first-order intensity statistics, three-dimensional shape descriptors, and gray-level co-occurrence matrix (GLCM) features. All available features from these classes were retained, resulting in 18 first-order features, 14 shape features, and 24 GLCM features, for a total of 56 radiomics features. The complete feature list is provided in Appendix C.

For the *i*-th nodule, the extracted features were arranged in a fixed order to form the radiomics vector

(3) $$f_{i}^{\mathrm{rad}}={\left[f_{i,1}^{\mathrm{rad}},f_{i,2}^{\mathrm{rad}},\ldots ,f_{i,56}^{\mathrm{rad}}\right]}^{⊤}\in R^{56}.$$

Diagnostic fields automatically generated by PyRadiomics were excluded. Feature names, ordering, and dimensionality were kept identical across the training, validation, internal test, and external evaluation datasets.

To prevent information leakage, all 56 radiomics features were standardized using z-score normalization [20], with the mean and standard deviation estimated exclusively from the training subset. The same training-derived normalization parameters were applied unchanged to the validation set, internal test set, and LIDC-IDRI external evaluation set. No normalization parameters were estimated from the validation, test, or external evaluation data.

### 2.5. Multimodal Fusion Strategies

To investigate how deep image features and handcrafted radiomics features should be integrated, we evaluated five multimodal fusion strategies: direct concatenation (F1), MLP fusion (F2), gated fusion (F3), low-rank bilinear fusion (F4), and multi-token cross-modal attention (F5) [21]. All fusion variants used the same deep image features, standardized 56-dimensional radiomics features, 256-dimensional output representation, classification head, data split, and training protocol. The five fusion strategies are summarized in Table 2.

In F1, the global deep feature and radiomics vector were directly concatenated and projected to a 256-dimensional node representation. F2 further introduced a nonlinear MLP after feature concatenation. F3 used a learned sigmoid gate to adaptively regulate the contributions of the projected image and radiomics representations. F4 modeled multiplicative interactions between the two modalities using a parameter-efficient low-rank bilinear transformation [22].

The primary RGGA-Net architecture used F5, a multi-token cross-modal attention module [23]. For the *i*-th nodule, the final three-dimensional CNN feature map $F_{i}\in R^{C\times D\times H\times W}$ was processed by adaptive average pooling with an output size of 2×2×2 and reshaped into eight spatial image tokens. In parallel, each of the 56 standardized radiomics features was mapped to a 128-dimensional learnable token, producing a radiomics token matrix $R_{i}\in R^{56\times 128}$.

The spatial image tokens were used as queries, whereas the radiomics tokens were used as keys and values:

(4) $$Q_{i}=X_{i}W_{Q},\;K_{i}=R_{i}W_{K},\;V_{i}=R_{i}W_{V},$$

where $Q_{i}\in R^{8\times 128}$ and $K_{i},V_{i}\in R^{56\times 128}$. Four-head cross-modal attention was then applied:

(5) $$O_{i}=\mathrm{MHA}\left(Q_{i},K_{i},V_{i}\right).$$

The corresponding attention tensor had dimensions B×4×8×56, allowing each spatial image token to interact with individual radiomics descriptors rather than with a single globally pooled radiomics vector. The attention output was refined using a residual connection, layer normalization, and a feed-forward network. The eight refined tokens were subsequently averaged and concatenated with the global deep feature. A linear projection generated the final 256-dimensional node representation $h_{i}$ for graph construction and message passing.

The multi-token cross-modal attention module was retained in the primary RGGA-Net architecture because it represents the prespecified fusion design of the proposed framework and enables explicit interactions between spatial image tokens and individual radiomics descriptors. The additional F1–F4 variants were introduced as controlled fusion alternatives to examine whether the observed performance was specifically dependent on the attention operator.

Importantly, this comparison was not used for post hoc selection of the final model based on the internal test set. All five variants were evaluated under the same data split, output dimensionality, and training protocol. Their comparison was therefore interpreted as a sensitivity analysis of the fusion component rather than as an optimization procedure for selecting the numerically best test-set configuration.

### 2.6. Multi-Nodule Graph Construction Based on Radiomics Similarity

For each CT scan containing multiple nodules, we constructed a nodule-level graph to model inter-nodule relationships. Each nodule was represented by its standardized radiomics feature vector, and graph connectivity was determined in the radiomics feature space rather than by spatial coordinates [24]. The motivation was to connect nodules according to similarity in morphological and textural characteristics, thereby allowing the graph to capture semantic relationships between nodules.

Inter-nodule similarity was quantified using Euclidean distance between standardized radiomics feature vectors. Because all radiomics features were standardized using statistics derived from the training set, heterogeneous feature dimensions were placed on a comparable scale, making Euclidean distance a simple and reproducible measure of overall radiomics similarity.

Based on these distances, a *k*-nearest-neighbor graph was constructed for each CT scan. We used k=5 during validation and testing to preserve local radiomics neighborhoods while avoiding unnecessarily dense graph connectivity. During training, *k* was randomly sampled from {4,5,6} to reduce dependence on a single neighborhood size and improve robustness to small variations in graph connectivity.

For CT scans containing only one nodule $\left(N_{m}=1\right)$, no inter-nodule edges were constructed, and the model reduced to a non-graph feature-fusion classifier.

For each connected pair of nodules $\left(i_{m},j\right)$, a 4-dimensional edge attribute vector $e_{ij}$ was further constructed from their radiomics relationship, including feature-space distance and direction-related information. These edge attributes were subsequently provided to the RGGA layer as additional radiomics-aware information describing the relationship between connected nodules.

### 2.7. Radiomics-Gated Graph Attention Mechanism

**Figure 2:**
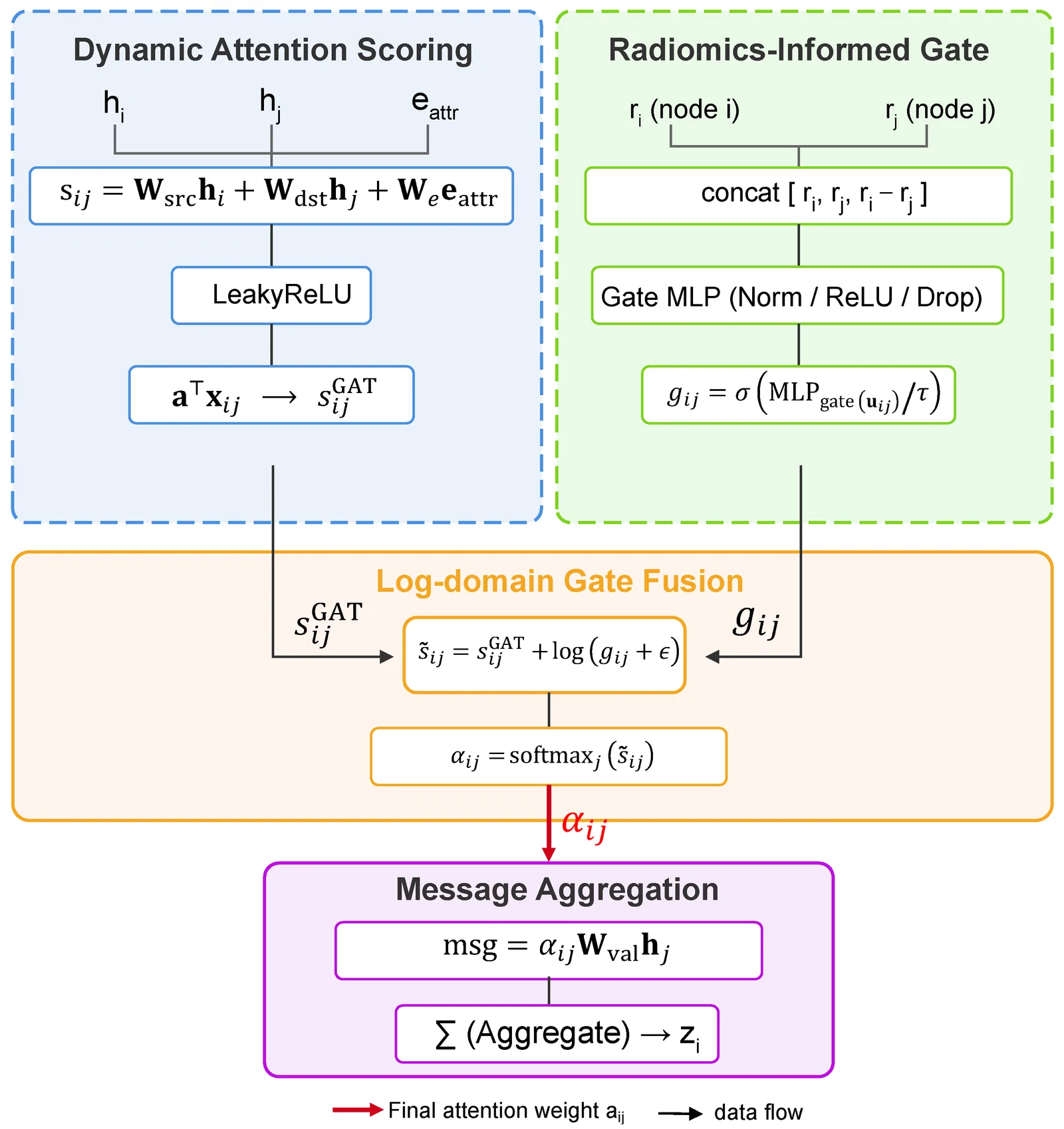
Architecture of the RGGA layer. The GATv2 branch computes a dynamic attention logit $s_{ij}^{\mathrm{GAT}}$ from node features $h_{i}$, $h_{j}$, and edge attribute $e_{ij}$ via linear projection, LeakyReLU, and attention-vector contraction. In parallel, the radiomics-informed gate concatenates the standardized radiomics vectors $r_{i}$, $r_{j}$, and their difference $r_{i}-r_{j}$, and passes them through a Gate MLP with a temperature-scaled sigmoid to produce a continuous gate value $g_{ij}$. The gate is incorporated into the attention logit through log-domain fusion, followed by softmax normalization over neighboring nodes to obtain the final attention coefficient $\alpha_{ij}$. Messages $\alpha_{ij}W_{\mathrm{val}}h_{j}$ are then aggregated to update the node embedding $z_{i}$.

The proposed graph learning module was built upon GATv2 [25,26] and further extended with a radiomics-guided gating mechanism [27,28]. The detailed architecture of the module is illustrated in Figure 2. For a target node *i* and its neighboring node *j*, the dynamic attention score is first computed as

(6) $$s_{ij}^{\mathrm{GAT}}=a^{⊤}e\mathrm{LeakyReLU}\left(W_{\mathrm{src}}h_{i}+W_{\mathrm{dst}}h_{j}+W_{e}e_{ij}\right),$$

where $h_{i}$ and $h_{j}$ denote the fused node features of nodules *i* and *j*, respectively; $e_{ij}$ denotes the edge attribute vector; $W_{\mathrm{src}}$, $W_{\mathrm{dst}}$, and $W_{e}$ are learnable projection matrices; and a is the attention vector. Here, $s_{ij}^{\mathrm{GAT}}$ is a scalar attention logit.

To incorporate radiomics information into graph message passing, we introduced a continuous edge gate derived from the radiomics representations of the two connected nodules. For an edge from node *j* to node *i*, the gate input was constructed as

(7) $$u_{ij}=r_{i}∥r_{j}∥\left(r_{i}-r_{j}\right),$$

where ∥ denotes vector concatenation and $r_{i}$ and $r_{j}$ are the standardized radiomics vectors of nodes *i* and *j*, respectively. The corresponding radiomics-guided gate was computed as

(8) $$g_{ij}=\sigma \left(\frac{{\mathrm{MLP}}_{\mathrm{gate}}\left(u_{ij}\right)}{\tau }\right),$$

where σ(·) is the sigmoid function and τ is a learnable temperature coefficient controlling the smoothness of the gate distribution. To ensure positivity during optimization, τ was parameterized as

(9) $$\tau =\mathrm{softplus}\left(\theta_{\tau }\right)+10^{-4},$$

where the unconstrained parameter $\theta_{\tau }$ was initialized to 0.5413, corresponding to an initial effective temperature of approximately 1.0001. This parameterization guarantees τ>0 throughout training. A smaller τ produces more polarized gate values, whereas a larger τ yields smoother intermediate values.

The scalar gate $g_{ij}\in \left(0,1\right)$ provides a continuous, radiomics-informed modulation of edge strength rather than performing binary edge selection. Edges considered more relevant by the gate receive greater message-passing weight, whereas edges assigned smaller gate values are relatively suppressed.

To avoid undesired effects caused by directly scaling signed attention logits, the radiomics-derived gate was incorporated in the logit space through additive log-domain fusion. Specifically,

(10) $${\tilde{s}}_{ij}=s_{ij}^{\mathrm{GAT}}+\log\left(g_{ij}+\epsilon \right),$$

where ϵ is a small positive constant for numerical stability. The normalized attention coefficient was then computed as

(11) $$\alpha_{ij}={\mathrm{softmax}}_{j}\left({\tilde{s}}_{ij}\right).$$

This formulation is equivalent to applying the learned gate as a multiplicative modulation in the unnormalized attention probability domain, while preserving the original ordering property of attention logits.

For multi-head graph attention, the output node embedding was obtained by aggregating messages from neighboring nodules:

(12) $$z_{i}=\overset{H}{\underset{m=1}{∥}}\sum_{j\in N(i)}\alpha_{ij}^{\left(m\right)}W_{v}^{\left(m\right)}h_{j},$$

where *H* denotes the number of attention heads, ∥ denotes concatenation across heads, $\alpha_{ij}^{\left(m\right)}$ is the normalized attention coefficient of the *m*-th head, and $W_{v}^{\left(m\right)}$ is the corresponding value projection matrix.

Two RGGA layers were stacked, with residual connections and layer normalization applied between successive layers. The resulting nodule-level embedding $z_{i}\in R^{256}$ was passed through a two-layer fully connected classification head implemented as

Linear(256,128)→ReLU→Dropout(0.1)→Linear(128,1).

The resulting scalar logit was converted into the malignancy probability as

(13) $${\hat{y}}_{i}=\sigma \left({\mathrm{MLP}}_{\mathrm{cls}}\left(z_{i}\right)\right).$$

For the non-graph fusion models, the same classifier structure was used, with the input dimension matched to the corresponding fused representation.

### 2.8. Joint Loss Function

The model was optimized using a joint objective consisting of focal loss [29] and anchor loss:

(14) $$L_{\mathrm{total}}=L_{\mathrm{Focal}}+\mu L_{\mathrm{Anchor}},$$

where μ controls the contribution of the anchor loss. The focal loss was used to alleviate the severe class imbalance between benign and malignant nodules.

For each nodule, the 56-dimensional standardized radiomics vector $r_{i}\in R^{56}$ was projected into the same 256-dimensional embedding space as the graph-learned node representation:

(15) $$z_{i}^{\mathrm{rad}}=W_{2}\mathrm{ReLU}\left(W_{1}r_{i}+b_{1}\right)+b_{2},$$

where $W_{1}\in R^{256\times 56}$ and $W_{2}\in R^{256\times 256}$. The resulting $z_{i}^{\mathrm{rad}}\in R^{256}$ served as the radiomics-guided reference representation.

The anchor loss was then defined as

(16) $$L_{\mathrm{Anchor}}=\mathrm{MSE}\left(z^{\mathrm{node}},z^{\mathrm{rad}}\right)=\frac{1}{N}\sum_{i=1}^{N}{∥z_{i}^{\mathrm{node}}-z_{i}^{\mathrm{rad}}∥}_{2}^{2}.$$

Here, $z_{i}^{\mathrm{node}}$ denotes the graph-learned node embedding, whereas $z_{i}^{\mathrm{rad}}$ denotes the corresponding radiomics-derived reference representation. Both representations are jointly optimized through the anchor objective, encouraging alignment between the graph-learned and radiomics-derived embedding spaces. The anchor-loss weight was set to μ=0.1 in the main configuration.

### 2.9. Experimental Settings and Statistical Analysis

All internal experiments were conducted using the leakage-free patient-level split described in Section 2.1.1. The internal held-out test set contained 870 nodules from 312 patients, including 94 malignant and 776 benign nodules. Each revised model was independently trained using the same three random seeds (2026, 2027, and 2028), and seed-specific results are reported as mean ± standard deviation (SD).

The principal model hyperparameters were selected based on validation-set performance and training stability. The internal held-out test set was not used for hyperparameter optimization or model selection. The final hyperparameter settings were kept fixed for test-set evaluation and are summarized in Table 3, with trainable parameter counts provided in Appendix B.

The primary discrimination metrics were the area under the receiver operating characteristic curve (AUC) and the area under the precision–recall curve (PR-AUC). Because malignant nodules accounted for 10.80% of the internal test set, PR-AUC was treated as a co-primary metric rather than as a secondary descriptive measure. Threshold-specific performance was evaluated using thresholds selected exclusively from the validation set. We report sensitivity, specificity, positive predictive value (PPV), negative predictive value (NPV), and F1 score at the validation-derived Youden operating point. A second operating point targeting approximately 90% sensitivity on the validation set was also evaluated. Probabilistic performance was assessed using the Brier score, calibration slope, calibration intercept, and expected calibration error (ECE).

For comparisons between models evaluated on the same test patients, paired patient-level bootstrap resampling was used to estimate differences in AUC and PR-AUC. All nodules belonging to a sampled patient were retained together during resampling. Confidence intervals that included zero were interpreted as insufficient evidence of a statistically robust performance difference.

To assess the potential influence of nodule-count-related shortcut information, we additionally evaluated a logistic-regression baseline using the number of annotated nodules within each CT examination as the sole predictor. The model was fitted on the LUNA25 training set and evaluated unchanged on the internal test set. We also performed an exploratory sensitivity analysis restricted to the N=2 subgroup, in which nodule count was constant, and compared RGGA + Anchor (G6) with the matched non-graph Fusion + Anchor model (G1) using paired patient-level bootstrap resampling.

## 3. Results

### 3.1. Comparative Experiments

To systematically evaluate the effectiveness of the proposed method, we compared RGGA-Net with several baseline models, including traditional radiomics-based models, deep learning models, and ablation variants of the proposed framework. These comparisons were designed to assess the contribution of multimodal feature fusion, graph-based inter-nodule relationship modeling, and the proposed radiomics-gated graph attention mechanism.

The compared methods are summarized in Table 4.

### 3.2. Internal Performance on the Test Set

Table 5 summarizes the internal test performance of the proposed model and the principal comparison methods under the patient-level split. All baseline models were evaluated under the same three-seed training protocol. Among the conventional baselines, the radiomics MLP performed well. In contrast, the GCN and 3D ResNet-18 showed lower performance.

The control experiments further confirmed the performance of the graph and fusion methods. The non-graph fusion model with anchor regularization achieved an AUC of 0.8703±0.0259 and a PR-AUC of 0.4795±0.0370. Meanwhile, GATv2 with anchor regularization remained stable, achieving an AUC of 0.8794±0.0334 and a PR-AUC of 0.4691±0.0695.

The complete RGGA-Net demonstrated the best overall performance. It achieved an AUC of 0.8910±0.0064 and a PR-AUC of 0.5173±0.0380. At the validation-derived Youden operating point, the model maintained a balanced profile. Its sensitivity, specificity, PPV, NPV, and F1 score were 0.9007±0.0546, 0.7844±0.0324, 0.3377±0.0216, 0.9851±0.0074, and 0.4902±0.0141, respectively. We also evaluated the model at an operating point targeting approximately 90% sensitivity on the validation set. Under this setting, the observed test sensitivity was 0.9043±0.0000. Additionally, the specificity was 0.7569±0.0470, the PPV was 0.3141±0.0394, the NPV was 0.9849±0.0010, and the F1 score was 0.4653±0.0441.

Compared with the strongest graph-based control, GATv2 with anchor regularization (GATv2 + Anchor), RGGA-Net increased the mean AUC by 0.0116 and the mean PR-AUC by 0.0482 across three random seeds. However, seed-specific paired patient-level bootstrap analysis revealed that the 95% confidence intervals for both ΔAUC and ΔPR-AUC included zero for all three seeds (shown as Appendix E). Therefore, although RGGA-Net achieves higher mean discrimination metrics and is highly competitive, the paired analysis does not provide statistically robust evidence to make a definitive superiority claim over GATv2 + Anchor. In the direct matched comparison with the non-graph Fusion + Anchor model (G1), RGGA-Net increased the mean AUC by 0.0207 and the mean PR-AUC by 0.0378 across the three random seeds. However, the seed-specific paired bootstrap 95% confidence intervals included zero for both metrics in all three seeds (shown as Appendix E), indicating that the incremental predictive benefit of graph message passing was not statistically established. Furthermore, RGGA-Net demonstrated distinct advantages over the strongest conventional baseline, the radiomics MLP; while it showed a smaller improvement in AUC, it provided a higher PR-AUC and better sensitivity-oriented performance. Ultimately, these results emphasize that performance differences should be interpreted using paired effect sizes and confidence intervals rather than relying on point estimates alone.

### 3.3. Calibration and Decision-Curve Analysis

**Figure 3:**
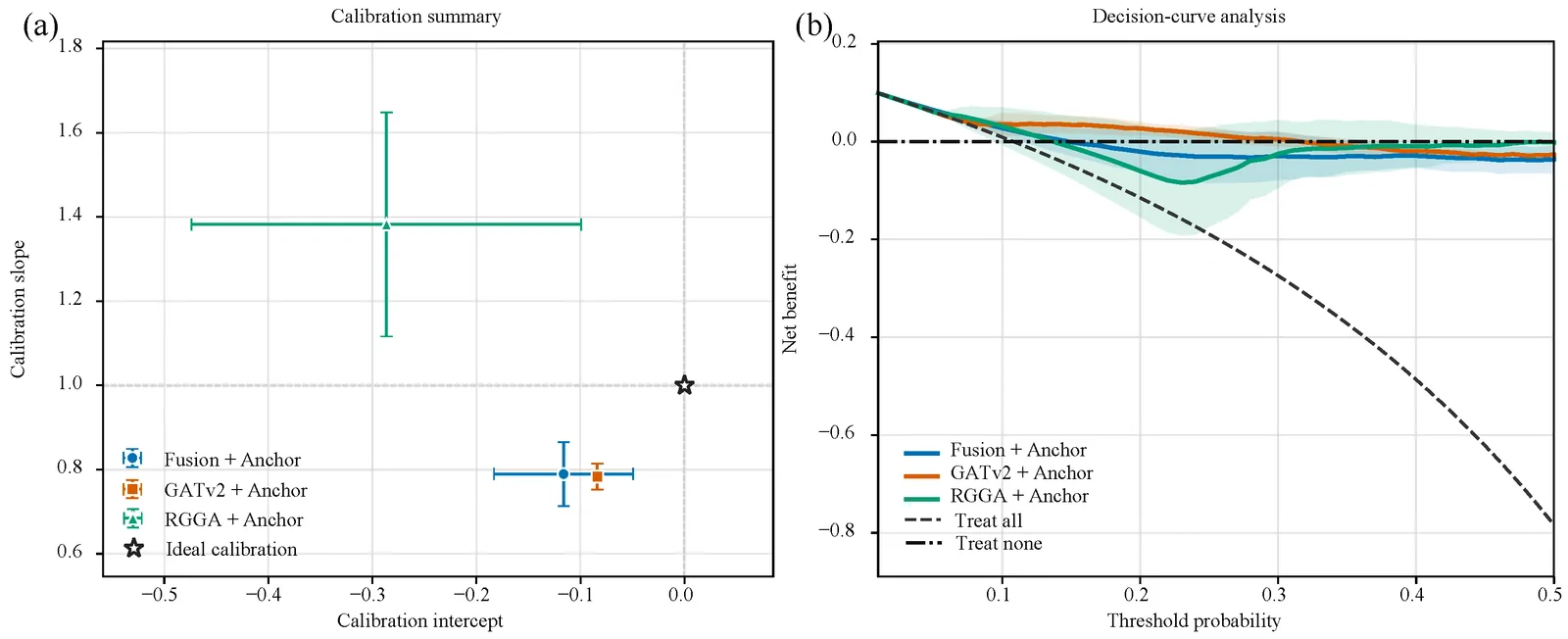
Calibration and decision-curve analysis on the internal held-out test set. (**a**) Calibration summary showing the mean calibration intercept and slope across three random seeds, with error bars indicating standard deviations. The star denotes the ideal calibration point (intercept =0, slope =1). (**b**) Decision-curve analysis showing the mean net benefit of Fusion + Anchor, GATv2 + Anchor, and RGGA-Net across threshold probabilities, together with the treat-all and treat-none reference strategies. Shaded regions indicate variability across the three random seeds.

The calibration results are summarized in Table 6 and Figure 3a. GATv2 with anchor regularization obtained the lowest mean Brier score and ECE among the three principal revised models. RGGA-Net achieved a calibration slope of 1.3824±0.2664, a calibration intercept of −0.2864±0.1872, and an ECE of 0.1734±0.1017. The slope above 1 and the relatively large seed-to-seed variability indicate that the gain in discrimination did not translate into uniformly improved probability calibration. Accordingly, RGGA-Net probabilities should not be interpreted as fully calibrated clinical risk estimates without additional external calibration.

As shown in Figure 3b, decision-curve analysis showed that RGGA-Net provided a positive mean net benefit relative to both the treat-none and treat-all strategies over a low-threshold interval of approximately 0.06–0.13. GATv2 with anchor regularization retained positive mean net benefit over a broader threshold interval in the current internal test set. These findings do not demonstrate a uniform decision-utility advantage for RGGA-Net and are therefore interpreted as descriptive rather than as evidence of clinical deployment readiness.

### 3.4. Controlled Comparison of Multimodal Fusion Strategies

**Figure 4:**
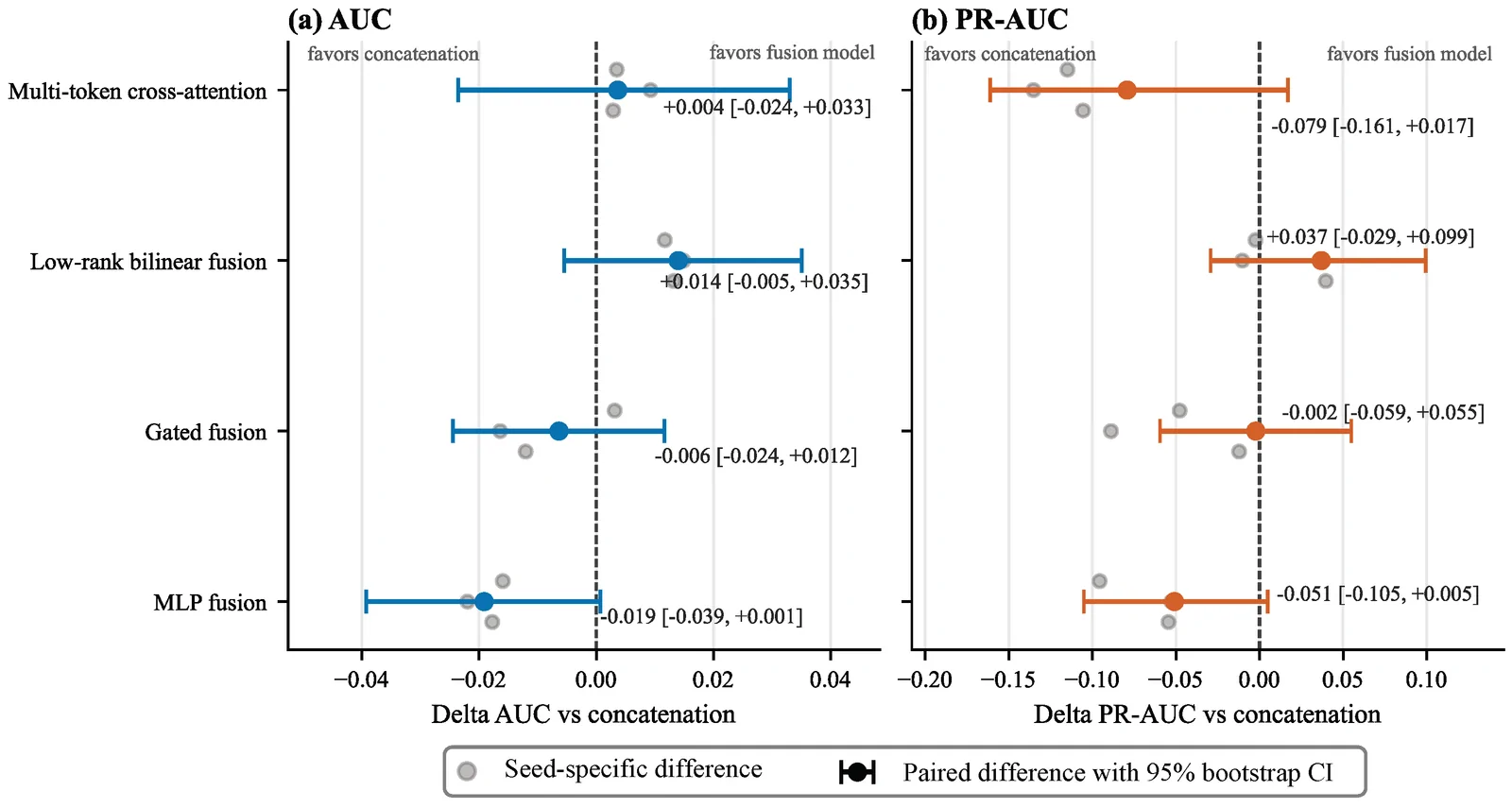
Controlled comparison of multimodal fusion strategies on the internal held-out test set. Paired differences in (**a**) AUC and (**b**) PR-AUC are reported relative to direct concatenation. Gray circles indicate seed-specific differences across three random seeds, whereas colored circles show the reported paired differences and horizontal error bars represent patient-level bootstrap 95% confidence intervals. Positive values favor the alternative fusion strategy, whereas negative values favor direct concatenation. All confidence intervals crossed zero, indicating that none of the alternative fusion operators demonstrated a statistically robust advantage over direct concatenation.

To determine whether the internal performance depended specifically on the multi-token attention operator, five multimodal fusion strategies were evaluated under the same data split, 256-dimensional output representation, classification head, and training protocol. Their absolute performance is reported in Table 7, and the paired effect-size comparison relative to direct concatenation is shown in Figure 4.

Low-rank bilinear fusion (F4) achieved the highest point estimates, with an AUC of 0.8868±0.0021 and a PR-AUC of 0.5435±0.0094. Direct concatenation (F1) achieved an AUC of 0.8736±0.0014 and a PR-AUC of 0.5345±0.0177. Multi-token attention (F5) produced a comparable AUC of 0.8788±0.0029 but a lower PR-AUC of 0.4160±0.0114. MLP fusion and gated fusion did not improve the mean discrimination metrics relative to direct concatenation.

As shown in Figure 4, the paired bootstrap confidence intervals for the differences relative to F1 included zero. Thus, the controlled analysis did not establish a statistically robust advantage for any individual fusion operator. F5 was retained in the primary RGGA-Net architecture because it was the prespecified fusion design and explicitly models interactions between spatial image tokens and individual radiomics descriptors. The F1–F4 experiments were treated as a sensitivity analysis of the fusion component rather than as a test-set-based procedure for retrospectively selecting the numerically best architecture.

### 3.5. Ablation Study and Gate–Anchor Interaction Analysis

Table 8 presents the stepwise ablation of the main RGGA-Net components. Experiment E2 uses the multi-token image–radiomics fusion without graph modeling. Compared with the CNN-only baseline (E1), introducing fusion substantially improved the test AUC. This gain suggests that the radiomics features provide complementary information beyond the image encoder alone. However, adding standard GATv2 message passing (E3) or the RGGA gate alone (E4) did not improve the mean AUC over E2. The complete model with anchor regularization (E5) achieved the highest AUC among all variants. This indicates that the learned gate is most effective when paired with the embedding constraint. Overall, the performance gain of RGGA-Net stems from the joint optimization of graph-based interaction modeling and anchor regularization, rather than graph propagation alone.

To separate the effects of graph attention, radiomics gating, and anchor regularization, Table 9 reports the extended G1–G6 controls. G1 represents the non-graph fusion model with anchor regularization, G2 and G3 compare standard GATv2 without and with anchor regularization, respectively, and G5 and G6 provide the corresponding comparison for the learned RGGA gate. G4 replaced the learnable radiomics-guided gate with a fixed distance-based gate.

**Figure 5:**
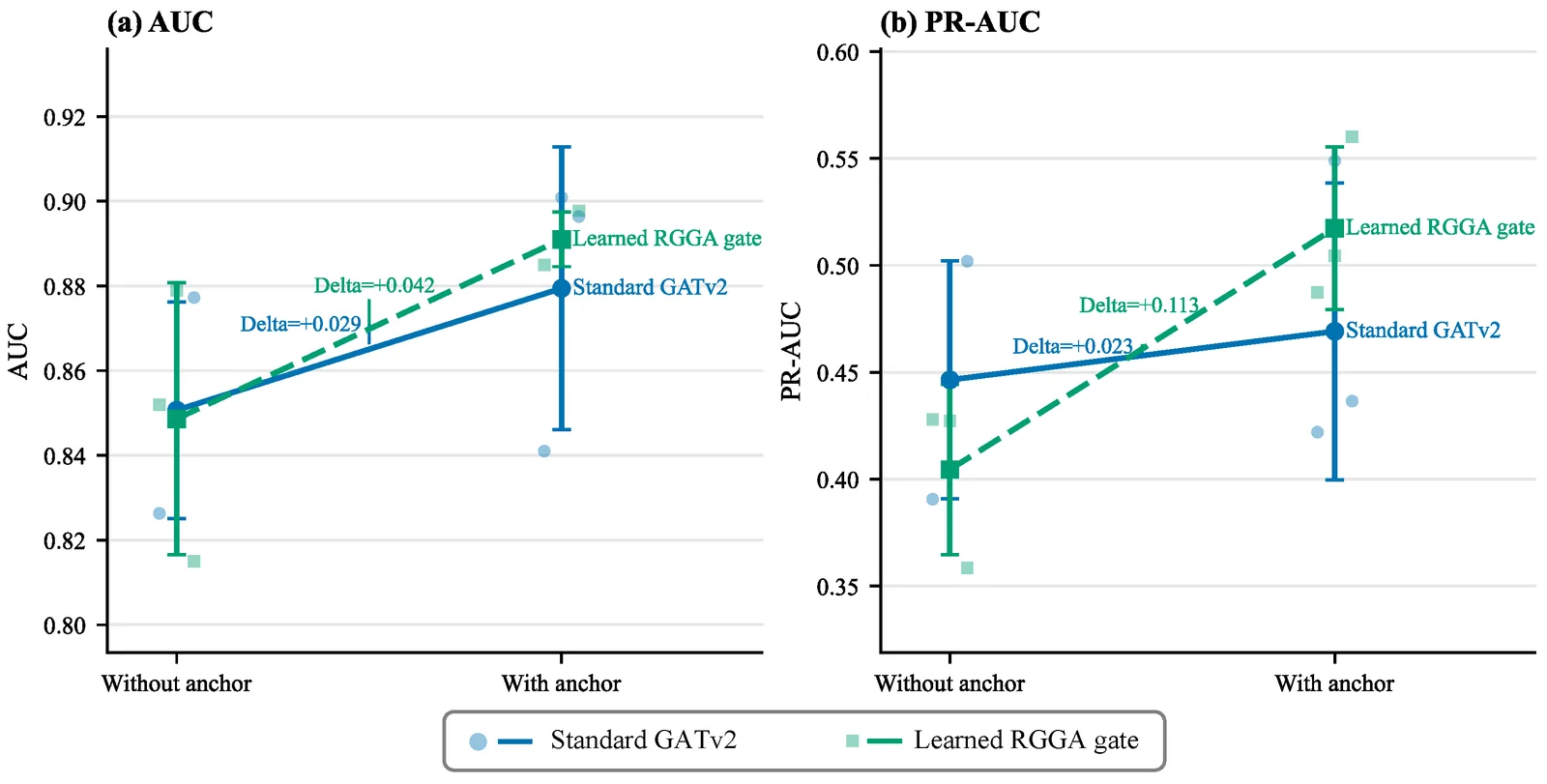
Ablation analysis of radiomics-guided gating and anchor regularization on the internal held-out test set. Panels (**a**,**b**) show the AUC and PR-AUC, respectively, of standard GATv2 and the learned RGGA gate, evaluated with and without anchor regularization. Large markers and error bars represent the mean and standard deviation across three random seeds, whereas lighter markers indicate seed-specific results. The annotated Δ values denote the mean performance change associated with adding anchor regularization. Anchor regularization produced a larger mean improvement for the learned RGGA gate, particularly in PR-AUC, suggesting a favorable interaction pattern; however, this result does not establish statistically significant synergy.

The learned gate without anchor regularization (G5) did not outperform standard GATv2 (G2). Adding anchor regularization increased the mean AUC and PR-AUC by 0.0287 and 0.0227 for GATv2, and by 0.0424 and 0.1128 for RGGA, respectively. As illustrated in Figure 5, this larger improvement indicates a favorable Gate–Anchor interaction pattern, but not statistically established synergy. GATv2 + Anchor achieved the lowest Brier score, whereas RGGA + Anchor achieved the highest AUC and PR-AUC.

A nodule-count-only logistic-regression baseline achieved an AUC of 0.7450 and a PR-AUC of 0.1939 on the internal test set, indicating that nodule count alone contained substantial predictive information in this cohort. In the exploratory analysis restricted to the N=2 subgroup, RGGA + Anchor showed a modest numerical improvement over Fusion + Anchor, with mean ΔAUC = 0.030 and mean ΔPR-AUC = 0.040. However, the paired 95% confidence intervals included zero. Given that this subgroup contained only 12 malignant nodules, these results should be interpreted as exploratory.

### 3.6. Cross-Dataset External Evaluation on LIDC-IDRI

Following multi-reader annotation aggregation, consensus-mask construction, cross-dataset duplicate screening, radiomics extraction, and quality control, the LIDC-IDRI cohort comprised 875 patients, 883 CT series, and 2651 nodule clusters. Under the primary strict label definition, 616 nodules were eligible for performance evaluation, including 315 benign and 301 malignant nodules. The remaining 2035 nodules had indeterminate strict labels and were excluded from metric calculation, although eligible indeterminate nodules were retained as contextual graph nodes when they passed preprocessing and quality-control requirements.

All LIDC-IDRI predictions were generated using models trained exclusively on LUNA25. The image preprocessing settings, radiomics feature schema, training-set standardization parameters, model weights, and classification thresholds derived from LUNA25 were transferred without modification. No model training, radiomics scaling, temperature fitting, threshold selection, or hyperparameter adjustment was performed using LIDC-IDRI.

The primary external comparison included Fusion + Anchor, GATv2 + Anchor, and RGGA + Anchor. As shown in Table 10, RGGA + Anchor achieved the highest AUC of 0.7023, compared with 0.6721 for GATv2 + Anchor and 0.6442 for Fusion + Anchor. In contrast, Fusion + Anchor achieved the highest PR-AUC of 0.7165, followed by RGGA + Anchor at 0.7000 and GATv2 + Anchor at 0.6921. GATv2 + Anchor achieved the lowest Brier score of 0.2353, whereas the corresponding values for RGGA + Anchor and Fusion + Anchor were 0.2504 and 0.2722, respectively. Thus, the higher AUC of RGGA + Anchor did not translate into uniformly better precision–recall performance or probability accuracy.

At the operating thresholds transferred from the LUNA25 validation set, the three models showed substantially different sensitivity–specificity profiles. Fusion + Anchor achieved a sensitivity of 0.9302 but a specificity of only 0.0952. GATv2 + Anchor achieved a sensitivity of 0.8704 and a specificity of 0.3016. RGGA + Anchor produced a specificity of 0.9556 and a PPV of 0.7879, but its sensitivity decreased to 0.1728, resulting in an F1 score of 0.2834. For RGGA + Anchor, the corresponding confusion matrix contained 301 true negatives, 14 false positives, 249 false negatives, and 52 true positives. These findings indicate that the discrimination measured across all possible thresholds transferred more reliably than the prespecified classification operating point.

Patient-level paired bootstrap analysis is summarized in Table 11. Relative to GATv2 + Anchor, the paired-bootstrap mean differences were 0.0308 for AUC (95% CI: −0.0175 to 0.0785) and 0.0082 for PR-AUC (95% CI: −0.0332 to 0.0492). Relative to Fusion + Anchor, the corresponding mean differences were 0.0589 for AUC (95% CI: −0.0090 to 0.1260) and −0.0154 for PR-AUC (95% CI: −0.0791 to 0.0495). Because all confidence intervals included zero, the external analysis did not establish a statistically robust advantage of RGGA + Anchor over either comparison model.

The external results were also sensitive to the definition used to binarize the radiologist-assigned malignancy scores. Under the primary strict median-score definition, RGGA + Anchor achieved an AUC of 0.7023 and a PR-AUC of 0.7000 on 616 nodules. Under the legacy mean-score definition, which included 1625 nodules comprising 1123 benign and 502 malignant cases, its AUC and PR-AUC decreased to 0.6519 and 0.4916, respectively. Because these label definitions produced different sample sizes and class prevalences, their PR-AUC values are not directly comparable. Nevertheless, the variation indicates that the estimated external performance depends partly on the LIDC-IDRI label-construction rule.

Overall, the frozen LUNA25-trained models retained moderate discrimination under the combined imaging-domain and reference-standard shifts represented by LIDC-IDRI. However, the transferred operating thresholds produced unstable sensitivity–specificity trade-offs, and the results varied according to the malignancy-score binarization rule. Because the LIDC-IDRI labels used here were derived from radiologist-assigned malignancy-suspicion scores, whereas LUNA25 used an outcome-based malignancy reference standard, this experiment is interpreted as cross-dataset external evaluation rather than clinical validation under an equivalent reference standard.

## 4. Discussion

In this study, we proposed RGGA-Net, a multimodal graph-based framework for pulmonary nodule malignancy classification. To address the limitation of conventional approaches that focus mainly on individual nodules, our model integrates deep imaging features, radiomics representations, and inter-nodule relationships. By combining cross-modal feature interaction with radiomics-guided graph reasoning, RGGA-Net captures complementary information from different domains. The cross-modal module enables information exchange between deep features and radiomics, allowing handcrafted quantitative descriptors to contribute beyond simple feature concatenation. Furthermore, the graph module incorporates relationships among nodules within the same CT examination, which leverages additional contextual information compared to analyzing nodules independently. Within this module, a radiomics-guided gate adapts graph message propagation through log-domain fusion, modulating edge contributions in the attention probability domain. Although the gate adaptively weights edges based on radiomics features, its value is learned by the neural network and should not be strictly interpreted as a predefined radiomics similarity measure.

Our ablation experiments and model comparisons demonstrate that individual components provide distinct benefits, rather than uniformly improving all metrics. The radiomics MLP remained a strong non-graph baseline, while GATv2 with anchor regularization achieved favorable calibration but a lower mean PR-AUC than RGGA-Net. Notably, using the radiomics gate alone did not consistently improve upon standard GATv2. However, combining the gate with anchor regularization achieved the highest mean AUC and PR-AUC. This observation suggests that embedding regularization helps stabilize graph-based feature propagation, highlighting a complementary interaction between graph modulation and representation regularization. Nevertheless, we must interpret these improvements cautiously. Because the paired confidence intervals overlapped among different variants, these results indicate a favorable interaction pattern rather than definitive statistical superiority. Similarly, the direct G6-versus-G1 comparison did not establish a statistically robust incremental benefit of graph message passing, despite higher mean AUC and PR-AUC for RGGA-Net.

The performance difference between ROC-AUC and PR-AUC requires careful interpretation, particularly given the marked class imbalance in the internal test set where only 10.80% of the nodules were malignant. Although RGGA-Net achieved a relatively high ROC-AUC, its PR-AUC remained modest. At the validation-derived Youden operating point, the model maintained high sensitivity but yielded only a moderate positive predictive value (PPV), indicating that false-positive predictions remain a major source of error. Because PR-AUC is highly sensitive to this behavior when positive cases are rare, ROC-AUC and PR-AUC must be evaluated together. A high ROC-AUC alone does not guarantee strong clinical performance in an imbalanced setting.

During the external evaluation on the LIDC-IDRI dataset, RGGA-Net achieved the highest AUC among all evaluated models. However, the paired bootstrap confidence intervals still included zero when compared to competing approaches. These findings suggest a moderate discriminative ability across different datasets, leaving the relative advantage of RGGA-Net over other models uncertain. Ultimately, this external test provides valuable insight into how the model behaves under varying imaging characteristics and malignancy reference standards.

The distribution of nodule counts remains an important consideration when interpreting the graph-based results. The nodule-count-only baseline achieved an AUC of 0.7450, confirming that nodule multiplicity itself carries substantial predictive information in the current cohort. Although the N=2 sensitivity analysis controlled for this factor and showed a modest numerical advantage for RGGA-Net, the confidence intervals included zero and only 12 malignant nodules were available in this subgroup. The association between nodule count and malignancy risk may also be cohort dependent: lower nodule count was associated with higher malignancy probability in the PanCan/Brock model [33], whereas the NELSON study found no significant difference in participant-level lung cancer probability across baseline nodule-count categories [34]. Therefore, the present analyses quantify but do not exclude nodule-count-related shortcut learning.

### Limitations

Several limitations must be acknowledged. First, the study relies entirely on retrospective datasets. Although we applied patient-level splitting internally, external generalizability remains a concern because differences in imaging protocols and malignancy reference standards can affect performance. Therefore, extensive multi-center validation is essential. Second, graph-based reasoning relies on the presence of multiple nodules in a single examination, so its usefulness for isolated nodules requires further investigation. Third, our graph construction depends on radiomics-derived phenotypic similarity and does not explicitly use anatomical locations or spatial relationships, such as lobar distribution or bilateral patterns. While radiomics similarity provides relational context, future models should integrate it with spatial coordinates and anatomical locations for a unified representation. Fourth, graph topology and edge weighting are vulnerable to segmentation variability and scanner-induced radiomics shifts. We did not perform dedicated perturbation or harmonization analyses, which will be crucial for future multi-center studies. Finally, incorporating longitudinal imaging changes and additional clinical data in future work will help provide a more comprehensive malignancy assessment.

## 5. Conclusions

RGGA-Net integrates cross-modal feature interaction, radiomics-guided graph reasoning, learned edge modulation, and anchor regularization for pulmonary nodule malignancy classification. The proposed framework extends conventional nodule-level analysis by incorporating inter-nodule contextual information. Internal evaluation on the LUNA25 cohort demonstrated promising discriminative performance, while external evaluation on LIDC-IDRI showed moderate cross-dataset generalization under imaging-domain and reference-standard shifts. However, the matched graph-versus-non-graph comparisons did not establish a statistically robust incremental predictive benefit of graph message passing. These results support RGGA-Net as a feasible framework for incorporating radiomics-guided inter-nodule context, although further validation on larger and prospective multi-center cohorts is required before clinical application.

## Figures and Tables

**Table 1:** Patient-level data split and nodule-count composition of the LUNA25 cohort. All CT scans and nodules belonging to the same patient were assigned to a single subset. In Panel B, values are reported as the total number of nodule samples, followed by the number and percentage of malignant nodules in parentheses. *N* denotes the number of nodules within the same CT scan.

(**A**) Overall data split						
**Subset**	**Patients**	**CT scans**	**Nodules**	**Malignant**	**Benign**	**Positive ratio**
Training	1469	2844	4289	362	3927	8.44%
Validation	315	590	888	83	805	9.35%
Test	312	583	870	94	776	10.80%
Total	2096	4017	6047	539	5508	8.91%
(**B**) Distribution by the number of nodules per CT scan						
**Subgroup**	**Training**		**Validation**		**Test**	
	**Nodules**	**Malignant**	**Nodules**	**Malignant**	**Nodules**	**Malignant**
N=1	1969	328 (16.66%)	399	77 (19.30%)	409	82 (20.05%)
N=2	1080	34 (3.15%)	254	6 (2.36%)	224	12 (5.36%)
N≥3	1240	0 (0.00%)	235	0 (0.00%)	237	0 (0.00%)

**Table 2:** Multimodal fusion strategies evaluated in the controlled fusion comparison. All variants produced a 256-dimensional fused representation and used the same classification head, data split, and training protocol.

Variant	Fusion Strategy	Description
F1	Concatenation	Direct concatenation followed by linear projection
F2	MLP fusion	Nonlinear fusion of concatenated image and radiomics features
F3	Gated fusion	Learned sigmoid gate for adaptive modality weighting
F4	Low-rank bilinear fusion	Parameter-efficient multiplicative cross-modal interaction
F5	Multi-token attention	Attention between 8 image tokens and 56 radiomics tokens

**Table 3:** Principal hyperparameter settings of RGGA-Net.

Hyperparameter	Value
Number of kNN neighbors	k=5 during validation and testing; k∼U{4,5,6} during training
Embedding dimension	256
Number of graph-attention heads	4
Number of graph layers	2
Anchor-loss weight μ	0.1
Focal-loss parameters γ/α	2/0.75
Random seeds	2026, 2027, and 2028

**Table 4:** Summary of compared methods for static malignancy risk assessment.

Method	Description	Type
Radiomics-only MLP [30]	A three-layer multilayer perceptron using only radiomics features as input	External baseline
3D ResNet-18 [6]	A standard 3D convolutional neural network for independent single-nodule prediction without graph structure or radiomics features	External baseline
CNN-only	The proposed 3D CNN backbone for independent single-nodule prediction	Ablation
CNN + Radiomics (w/o graph) [9]	Fusion of deep features and radiomics features without graph modeling	Ablation
3D Swin Transformer [7]	A 3D Swin Transformer-based baseline for independent single-nodule prediction, leveraging hierarchical self-attention to capture long-range dependencies in volumetric CT data	External baseline
GCN [31,32]	A standard graph convolutional network applied to the same multimodal fused features as our model, without radiomics-guided gating or attention mechanisms	External baseline
GATv2 [11]	Standard GATv2 applied to fused node features without the proposed radiomics-guided gating mechanism	Baseline & Ablation
**RGGA-Net**	Full model with multimodal feature fusion, radiomics-similarity-based graph construction, radiomics-gated graph attention, and anchor loss	**Proposed**

**Note:** The bold method name denotes our proposed method.

**Table 5:** Internal held-out test performance under the patient-level split. Values are reported as mean ± SD across three random seeds. Threshold-specific metrics were calculated using validation-derived Youden thresholds.

Method	AUC	PR-AUC	Sensitivity	Specificity	PPV	NPV	F1	Brier
Radiomics MLP	0.8603±0.0021	0.5053±0.0129	0.7730±0.1429	0.7809±0.1017	0.3129±0.0547	0.9677±0.0182	0.4382±0.0398	0.1420±0.0084
3D ResNet-18	0.5896±0.0933	0.1457±0.0378	0.4752±0.2248	0.6151±0.1463	0.1309±0.0325	0.9090±0.0262	0.2012±0.0489	0.1597±0.0841
3D Swin Transformer	0.7235±0.0418	0.2867±0.0524	0.6915±0.0536	0.7466±0.0197	0.2492±0.0292	0.9522±0.0090	0.3663±0.0390	0.1358±0.0187
GCN	0.7972±0.0722	0.4219±0.0952	0.6383±0.1151	0.8419±0.0134	0.3284±0.0585	0.9505±0.0157	0.4337±0.0775	0.1162±0.0088
Fusion + Anchor (G1)	0.8703±0.0259	0.4795±0.0370	0.8191±0.0106	0.7749±0.0674	0.3138±0.0560	0.9725±0.0012	0.4516±0.0591	0.1101±0.0216
GATv2 + Anchor (G3)	0.8794±0.0334	0.4691±0.0695	0.8972±0.0503	0.7169±0.0345	0.2790±0.0327	0.9828±0.0089	0.4253±0.0423	0.0942±0.0081
**RGGA-Net (G6)**	0.8910±0.0064	0.5173±0.0380	0.9007±0.0546	0.7844±0.0324	0.3377±0.0216	0.9851±0.0074	0.4902±0.0141	0.1088±0.0359

**Note:** The bold method name denotes our proposed method. Bold values denote the best performance per metric.

**Table 6:** Calibration performance on the internal held-out test set. Values are mean ± SD across three random seeds.

Model	Brier Score	Calibration Slope	Calibration Intercept	ECE
Fusion + Anchor (G1)	0.1101±0.0216	0.7895±0.0758	−0.1162±0.0669	0.1732±0.0593
GATv2 + Anchor (G3)	0.0942±0.0081	0.7832±0.0308	−0.0838±0.0023	0.1372±0.0120
RGGA-Net (G6)	0.1088±0.0359	1.3824±0.2664	−0.2864±0.1872	0.1734±0.1017

**Table 7:** Controlled comparison of multimodal fusion strategies on the internal held-out test set. Values are mean ± SD across three random seeds.

Variant	Fusion Strategy	AUC	PR-AUC	Brier Score
F1	Direct concatenation	0.8736±0.0014	0.5345±0.0177	0.1148±0.0028
F2	MLP fusion	0.8550±0.0037	0.4669±0.0231	0.1032±0.0012
F3	Gated fusion	0.8651±0.0099	0.4849±0.0246	0.1237±0.0023
F4	Low-rank bilinear fusion	0.8868±0.0021	0.5435±0.0094	0.1176±0.0004
F5	Multi-token attention	0.8788±0.0029	0.4160±0.0114	0.1070±0.0010

**Note:** Bold values denote the best performance per metric.

**Table 8:** Stepwise ablation study of RGGA-Net on the internal held-out test set. Values are reported as mean ± SD across three random seeds. E2 uses the multi-token image–radiomics fusion module without graph message passing.

Experiment	Fusion	Graph	RGGA Gate	Anchor Loss	Test AUC	Δ vs. E1
E1: CNN-only	×	×	×	×	0.5842±0.0626	–
E2: CNN + Radiomics (w/o graph)	✓	×	×	×	0.8788±0.0029	+0.2946
E3: GATv2	✓	✓	×	×	0.8507±0.0255	+0.2665
E4: RGGA w/o anchor loss	✓	✓	✓	×	0.8486±0.0321	+0.2644
E5: Full RGGA-Net	✓	✓	✓	✓	0.8910±0.0064	+0.3068

**Note:** Bold values denote the best performance per metric.

**Table 9:** Extended analysis of graph attention, radiomics gating, and anchor regularization on the internal held-out test set. Values are mean ± SD across three random seeds. Lower Brier scores indicate better probability accuracy.

Variant	Model	Graph Module	Edge Modulation	Anchor Loss	AUC	PR-AUC	Brier Score
G1	Fusion + Anchor	None	None	Yes	0.8703±0.0259	0.4795±0.0370	0.1101±0.0216
G2	GATv2	GATv2	None	No	0.8507±0.0255	0.4464±0.0557	0.1109±0.0168
G3	GATv2 + Anchor	GATv2	None	Yes	0.8794±0.0334	0.4691±0.0695	0.0942±0.0081
G4	Fixed Gate + Anchor	GATv2	Fixed	Yes	0.8790±0.0077	0.4703±0.0450	0.1220±0.0118
G5	RGGA	GATv2	Learned	No	0.8486±0.0321	0.4045±0.0399	0.1395±0.0570
G6	**RGGA + Anchor**	GATv2	Learned	Yes	0.8910±0.0064	0.5173±0.0380	0.1088±0.0359

**Note:** The bold method name denotes our proposed method. Bold values denote the best performance per metric.

**Table 10:** Cross-dataset external evaluation on LIDC-IDRI using the primary strict label definition. Results were calculated from the three-seed ensemble probabilities. Threshold-specific metrics used the corresponding operating thresholds selected exclusively from the LUNA25 validation set.

Model	AUC	PR-AUC	Brier	Sensitivity	Specificity	PPV	NPV	F1
Fusion + Anchor	0.6442	**0.7165**	0.2722	**0.9302**	0.0952	0.4956	0.5882	0.6467
GATv2 + Anchor	0.6721	0.6921	**0.2353**	0.8704	0.3016	0.5436	**0.7090**	**0.6692**
**RGGA + Anchor**	**0.7023**	0.7000	0.2504	0.1728	**0.9556**	**0.7879**	0.5473	0.2834

**Note:** The bold method name denotes our proposed method. Bold values denote the best performance per metric.

**Table 11:** Patient-level paired bootstrap differences between RGGA + Anchor and the comparison models on LIDC-IDRI. Positive values favor RGGA + Anchor. All confidence intervals included zero.

Comparison	ΔAUC (95% CI)	ΔPR-AUC (95% CI)
RGGA vs. GATv2 + Anchor	0.0308 (−0.0175, 0.0785)	0.0082 (−0.0332, 0.0492)
RGGA vs. Fusion + Anchor	0.0589 (−0.0090, 0.1260)	−0.0154 (−0.0791, 0.0495)

**Figure A1:**
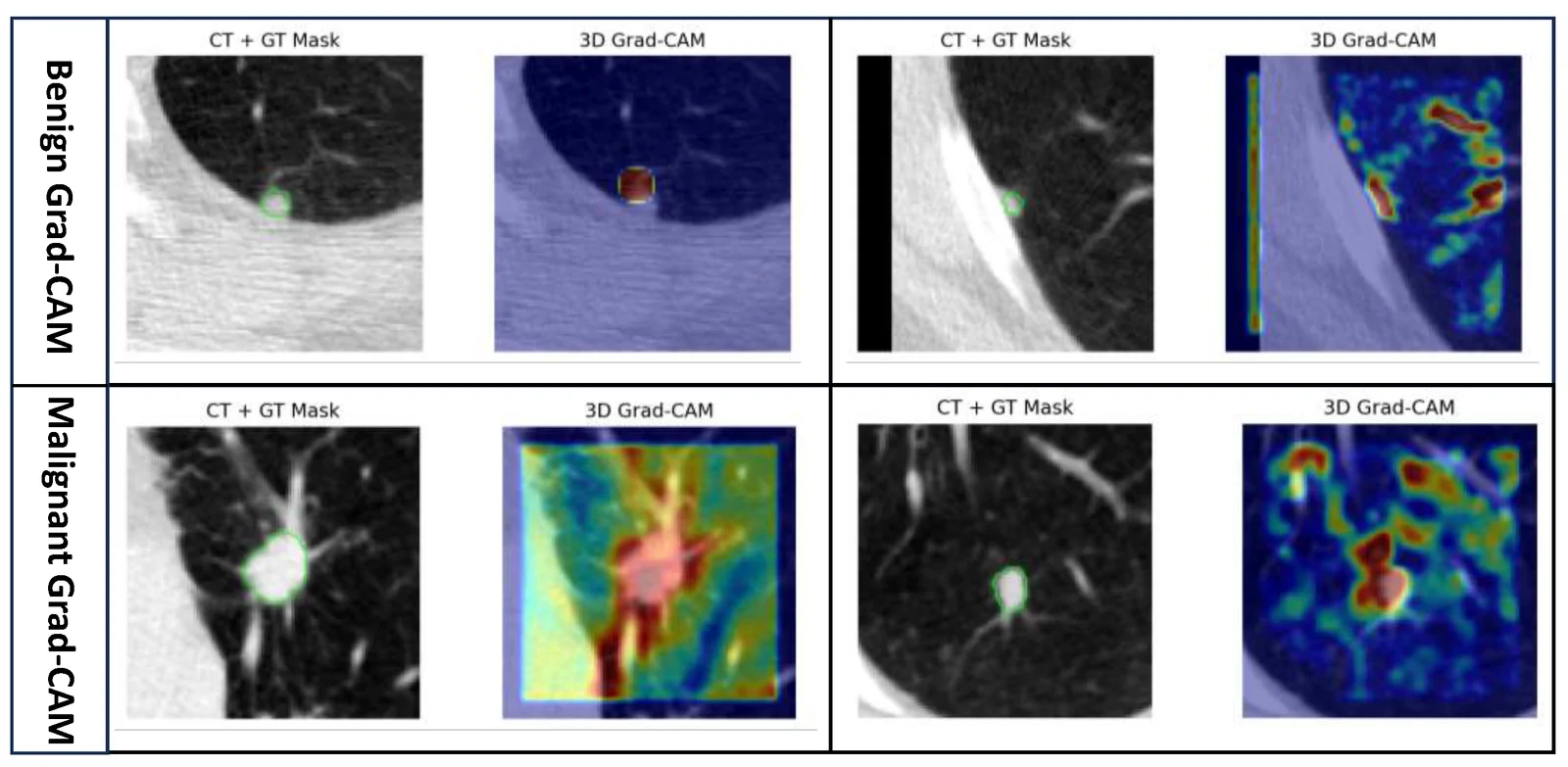
Representative 3D Grad-CAM visualizations for benign and malignant pulmonary nodules. Each example shows the CT image with the ground-truth nodule contour, the Grad-CAM activation overlay, and a magnified visualization of the nodule region. Warmer colors indicate regions with greater contribution to the corresponding node-level prediction. The visualizations are provided for qualitative interpretation and should not be considered anatomical localization or segmentation results.

## Data Availability

The data analyzed in this study are publicly available. The LUNA25 imaging data are available on Zenodo at https://zenodo.org/records/14223624 (accessed on 26 August 2026), and the corresponding annotation data are available at https://zenodo.org/records/14673658 (accessed on 26 August 2026). The LIDC-IDRI dataset is publicly available from The Cancer Imaging Archive at https://doi.org/10.7937/K9/TCIA.2015.LO9QL9SX (accessed on 26 August 2026).

## Abbreviations

The following abbreviations are used in this manuscript:

AUC	Area under the receiver operating characteristic curve
CI	Confidence interval
CNN	Convolutional neural network
CT	Computed tomography
GAT	Graph attention network
GATv2	Graph attention network version 2
GCN	Graph convolutional network
GLCM	Gray-level co-occurrence matrix
GNN	Graph neural network
kNN	k-nearest neighbor
LDCT	Low-dose computed tomography
LUNA25	Lung nodule analysis 2025 challenge
MedSAM2	Medical segment anything model 2
MHA	Multi-head attention
MLP	Multilayer perceptron
NLST	National Lung Screening Trial
PR-AUC	Area under the precision–recall curve
RGGA	Radiomics-gated graph attention
ROI	Region of interest
LIDC-IDRI	Lung Image Database Consortium and Image Database Resource Initiative

## Appendix A. Network Architecture Details

**Table A1:** Detailed configuration of the 3D CNN feature extractor.

Stage	Input Ch.	Output Ch.	Kernel	Stride	Padding	Pooling
Input	1	1	–	–	–	–
Block 1	1	32	3×3×3	1	1	2×2×2, stride 2
Block 2	32	64	3×3×3	1	1	2×2×2, stride 2
Block 3	64	128	3×3×3	1	1	2×2×2, stride 2
Block 4	128	256	3×3×3	1	1	2×2×2, stride 2
Global pooling	256	256	AdaptiveAvgPool3D(1,1,1)	–	–	–
Projection	256	256	Linear	–	–	–

## Appendix B. Parameter Counts of Active Model Components

**Table A2:** Parameter counts of the active model components in the evaluated configurations. The anchor projector is included only in configurations using anchor regularization; components not used in a given configuration are reported as zero.

Model	CNN Backbone	Fusion Module	Graph Module	Classifier	Anchor Projector	Total
F1_concat	1,229,312	146,432	0	33,025	0	1,408,769
F2_mlp	1,229,312	163,008	0	33,025	0	1,425,345
F3_gated	1,229,312	146,432	0	33,025	0	1,408,769
F4_bilinear	1,229,312	73,728	0	33,025	0	1,336,065
F5_token_attention	1,229,312	271,872	0	33,025	0	1,534,209
G1_anchor_only	1,229,312	271,872	0	33,025	80,384	1,614,593
G2_gatv2	1,229,312	271,872	1,316,096	33,025	0	2,850,305
G3_gatv2_anchor	1,229,312	271,872	1,316,096	33,025	80,384	2,930,689
G4_fixed_gate_anchor	1,229,312	271,872	854,656	33,025	80,384	2,469,249
G5_rgga	1,229,312	271,872	899,466	33,025	0	2,433,675
G6_rgga_anchor	1,229,312	271,872	899,466	33,025	80,384	2,514,059

## Appendix C. Complete Radiomics Feature List and Extraction Configuration

A total of 56 radiomics features were used in this study. The complete feature list is provided in Table A3.

**Table A3:** Complete list of the 56 radiomics features used in this study.

No.	Feature Class	Feature Name
1	Shape	original_shape_Elongation
2	Shape	original_shape_Flatness
3	Shape	original_shape_LeastAxisLength
4	Shape	original_shape_MajorAxisLength
5	Shape	original_shape_Maximum2DDiameterColumn
6	Shape	original_shape_Maximum2DDiameterRow
7	Shape	original_shape_Maximum2DDiameterSlice
8	Shape	original_shape_Maximum3DDiameter
9	Shape	original_shape_MeshVolume
10	Shape	original_shape_MinorAxisLength
11	Shape	original_shape_Sphericity
12	Shape	original_shape_SurfaceArea
13	Shape	original_shape_SurfaceVolumeRatio
14	Shape	original_shape_VoxelVolume
15	First-order	original_firstorder_10Percentile
16	First-order	original_firstorder_90Percentile
17	First-order	original_firstorder_Energy
18	First-order	original_firstorder_Entropy
19	First-order	original_firstorder_InterquartileRange
20	First-order	original_firstorder_Kurtosis
21	First-order	original_firstorder_Maximum
22	First-order	original_firstorder_MeanAbsoluteDeviation
23	First-order	original_firstorder_Mean
24	First-order	original_firstorder_Median
25	First-order	original_firstorder_Minimum
26	First-order	original_firstorder_Range
27	First-order	original_firstorder_RobustMeanAbsoluteDeviation
28	First-order	original_firstorder_RootMeanSquared
29	First-order	original_firstorder_Skewness
30	First-order	original_firstorder_TotalEnergy
31	First-order	original_firstorder_Uniformity
32	First-order	original_firstorder_Variance
33	GLCM	original_glcm_Autocorrelation
34	GLCM	original_glcm_ClusterProminence
35	GLCM	original_glcm_ClusterShade
36	GLCM	original_glcm_ClusterTendency
37	GLCM	original_glcm_Contrast
38	GLCM	original_glcm_Correlation
39	GLCM	original_glcm_DifferenceAverage
40	GLCM	original_glcm_DifferenceEntropy
41	GLCM	original_glcm_DifferenceVariance
42	GLCM	original_glcm_Id
43	GLCM	original_glcm_Idm
44	GLCM	original_glcm_Idmn
45	GLCM	original_glcm_Idn
46	GLCM	original_glcm_Imc1
47	GLCM	original_glcm_Imc2
48	GLCM	original_glcm_InverseVariance
49	GLCM	original_glcm_JointAverage
50	GLCM	original_glcm_JointEnergy
51	GLCM	original_glcm_JointEntropy
52	GLCM	original_glcm_MCC
53	GLCM	original_glcm_MaximumProbability
54	GLCM	original_glcm_SumAverage
55	GLCM	original_glcm_SumEntropy
56	GLCM	original_glcm_SumSquares

## Appendix D. 3D Grad-CAM Visualization

### Appendix D.1. Grad-CAM Generation and Processing

To provide qualitative visualization of the image regions contributing to nodule-level predictions, 3D Grad-CAM was applied to late convolutional layers of the CNN backbone. Two late convolutional layers, corresponding to the second- and third-last Conv3d layers, were examined as candidate target layers for qualitative visualization.

For a selected nodule, a forward pass was first performed using the trained model in evaluation mode to obtain the node-level classification logit. The malignant-class logit was used as the target score for malignant predictions, whereas its negative value was used for benign predictions. Activations A and gradients G were recorded from the selected convolutional layer, and Grad-CAM++-style channel weights were calculated as

(A1) $$\alpha =\frac{G^{2}}{2G^{2}+AG^{3}+\epsilon }.$$

The channel weights were obtained by spatially summing αReLU(G), and the resulting 3D activation map was computed as the ReLU of the weighted combination of feature maps. The CAM was then upsampled to the input CT patch size using trilinear interpolation.

To reduce activation artifacts near the patch boundary, a border-suppression mask was applied with a margin of

(A2) $$m=\max\left(2,\;\mathrm{round}\left[0.12\min(D,H,W)\right]\right),$$

where *D*, *H*, and *W* denote the CAM dimensions. The resulting CAM was normalized to [0,1] after percentile clipping at the 1st and 99th percentiles.

### Appendix D.2. Qualitative Interpretation

Representative 3D Grad-CAM visualizations are shown in Figure A1. The activation maps demonstrate that the CNN backbone responds to spatially localized image patterns within and around the pulmonary nodules, while the extent and distribution of activation vary across individual cases. Some examples show concentrated activation near the nodule region, whereas others exhibit more spatially diffuse responses.

These visualizations are intended as qualitative illustrations of model activation rather than quantitative evidence of anatomical localization. Because the classification network was not explicitly trained for lesion segmentation or localization, activation outside the annotated nodule should not be interpreted as evidence of tissue invasion or other specific pathological processes.

## Appendix E. Seed-Specific Paired Bootstrap Analysis

To further quantify the effects of graph attention, radiomics-guided gating, and anchor regularization, paired patient-level bootstrap analysis was performed for the principal ablation comparisons. Bootstrap resampling was conducted separately for each random seed, with all nodules belonging to the same patient retained together during resampling. Positive differences favor the first model listed in each comparison.

**Table A4:** Seed-specific paired patient-level bootstrap comparisons among the principal ablation variants on the internal held-out test set. G1 denotes Fusion + Anchor, G2 denotes GATv2, G3 denotes GATv2 + Anchor, G5 denotes RGGA without anchor regularization, and G6 denotes RGGA + Anchor.

Comparison	Seed	ΔAUC (95% CI)	ΔPR-AUC (95% CI)
G6–G1	2026	−0.0060(−0.0472,0.0306)	−0.0140(−0.0940,0.0645)
G6–G1	2027	0.0088(−0.0247,0.0366)	0.0039(−0.0777,0.0935)
G6–G1	2028	0.0578(−0.0017,0.1144)	0.1205(−0.0141,0.2455)
G6–G3	2026	0.0436 (−0.0167, 0.1119)	0.0640 (−0.0600, 0.1975)
G6–G3	2027	−0.0103 (−0.0420, 0.0145)	−0.0420 (−0.1303, 0.0367)
G6–G3	2028	0.0035 (−0.0601, 0.0604)	0.1162 (−0.0426, 0.2645)
G6–G2	2026	0.0557 (0.0016, 0.1164)	0.0937 (−0.0128, 0.2026)
G6–G2	2027	0.0412 (−0.0131, 0.0948)	0.0570 (−0.0316, 0.1394)
G6–G2	2028	0.0222 (−0.0334, 0.0770)	0.0592 (−0.0634, 0.2025)
G6–G5	2026	0.0315 (−0.0065, 0.0721)	0.0551 (−0.0390, 0.1551)
G6–G5	2027	0.0110 (−0.0417, 0.0537)	0.0720 (−0.0586, 0.1929)
G6–G5	2028	0.0849 (0.0165, 0.1409)	0.2012 (0.0552, 0.3416)
G3–G2	2026	0.0121 (−0.0437, 0.0712)	0.0297 (−0.0811, 0.1471)
G3–G2	2027	0.0515 (0.0007, 0.1052)	0.0990 (−0.0103, 0.2043)
G3–G2	2028	0.0188 (−0.0342, 0.0715)	−0.0570 (−0.1857, 0.0842)
G5–G2	2026	0.0242 (−0.0161, 0.0673)	0.0385 (−0.0510, 0.1251)
G5–G2	2027	0.0302 (−0.0295, 0.0951)	−0.0151 (−0.1674, 0.1462)
G5–G2	2028	−0.0627 (−0.0972, −0.0293)	−0.1420 (−0.2055, −0.0794)

## References

[1] The National Lung Screening Trial Research Team (2011). Reduced lung-cancer mortality with low-dose computed tomographic screening. N. Engl. J. Med..

[2] Ardila D., Kiraly A.P., Bharadwaj S., Choi B., Reicher J.J., Peng L., Tse D., Etemadi M., Ye W., Corrado G. (2019). End-to-end lung cancer screening with three-dimensional deep learning on low-dose chest computed tomography. Nat. Med..

[3] Wang C., Shao J., He Y., Wu J., Liu X., Yang L., Wei Y., Zhou X.S., Zhan Y., Shi F. (2024). Data-driven risk stratification and precision management of pulmonary nodules detected on chest computed tomography. Nat. Med..

[4] Tong G., Xue Z., Dang T., Cao T., Zhao X. Lung nodule malignancy classification on 3D CT images using a cosine similarity-enhanced graph attention network. Proceedings of the 2025 47th Annual International Conference of the IEEE Engineering in Medicine and Biology Society (EMBC).

[5] Venkadesh K.V., Setio A.A.A., Schreuder A., Scholten E.T., Chung K., W. Wille M.M., Saghir Z., Van Ginneken B., Prokop M., Jacobs C. (2021). Deep learning for malignancy risk estimation of pulmonary nodules detected at low-dose screening CT. Radiology.

[6] Wu P., Sun X., Zhao Z., Wang H., Pan S., Schuller B. (2020). Classification of Lung Nodules Based on Deep Residual Networks and Migration Learning. Comput. Intell. Neurosci..

[7] Zhao X., Xu J., Lin Z., Xue X. (2024). BiCFormer: Swin Transformer based model for classification of benign and malignant pulmonary nodules. Meas. Sci. Technol..

[8] Lin C.Y., Guo S.M., Lien J.J.J., Lin W.T., Liu Y.S., Lai C.H., Hsu I.L., Chang C.C., Tseng Y.L. (2023). Combined model integrating deep learning, radiomics, and clinical data to classify lung nodules at chest CT. Radiol. Med..

[9] Du L., Tang G., Che Y., Ling S., Chen X., Pan X. (2025). Fusing radiomics and deep learning features for automated classification of multi-type pulmonary nodule. Med. Phys..

[10] Deng H., Huang W., Zhou X., Zhou T., Fan L., Liu S. (2024). Prediction of benign and malignant ground glass pulmonary nodules based on multi-feature fusion of attention mechanism. Front. Oncol..

[11] Ma L., Wan C., Hao K., Cai A., Liu L. (2023). A novel fusion algorithm for benign-malignant lung nodule classification on CT images. BMC Pulm. Med..

[12] Bhise M.R., Ohmshankar S., Suchithra B., Archana C., Keerthana N.V. Lung nodule classification using transformer - depending on multi-view radiomics fusion. Proceedings of the 2025 2nd Asian Conference on Intelligent Technologies (ACOIT).

[13] Peeters D., Obreja B., Antonissen N., Saghir Z., Pastorino U., Silva M., de Bock G.H., Gietema H., Gleeson F., Heuvelmans M.A. (2026). Benchmarking of AI and Radiologists for Indeterminate Lung Nodule Malignancy Risk Estimation on Screening CT: The LUNA25 Challenge. Radiol. Artif. Intell..

[14] Faizi M.K., Qiang Y., Shagar M.M.B., Wei Y., Qiao Y., Zhao J., Urrehman Z. (2025). Graph neural network model using radiomics for lung CT image segmentation. Sci. Rep..

[15] Ma J., Yang Z., Kim S., Chen B., Baharoon M., Fallahpour A., Asakereh R., Lyu H., Wang B. (2025). MedSAM2: Segment Anything in 3D Medical Images and Videos. arXiv.

[16] Ma J., He Y., Li F., Han L., You C., Wang B. (2024). Segment anything in medical images. Nat. Commun..

[17] Armato S.G., McLennan G., Bidaut L., McNitt-Gray M.F., Meyer C.R., Reeves A.P., Zhao B., Aberle D.R., Henschke C.I., Hoffman E.A. (2011). The Lung Image Database Consortium (LIDC) and Image Database Resource Initiative (IDRI): A completed reference database of lung nodules on CT scans. Med. Phys..

[18] Van Griethuysen J.J., Fedorov A., Parmar C., Hosny A., Aucoin N., Narayan V., Beets-Tan R.G., Fillion-Robin J.C., Pieper S., Aerts H.J. (2017). Computational radiomics system to decode the radiographic phenotype. Cancer Res..

[19] Zwanenburg A., Vallières M., Abdalah M.A., Aerts H.J.W.L., Andrearczyk V., Apte A., Ashrafinia S., Bakas S., Beukinga R.J., Boellaard R. (2020). The Image Biomarker Standardization Initiative: Standardized Quantitative Radiomics for High-Throughput Image-Based Phenotyping. Radiology.

[20] Haga A., Takahashi W., Aoki S., Nawa K., Yamashita H., Abe O., Nakagawa K. (2019). Standardization of Imaging Features for Radiomics Analysis. J. Med. Investig..

[21] Huang B., Yang F., Yin M., Mo X., Zhong C. (2020). A review of multimodal medical image fusion techniques. Comput. Math. Methods Med..

[22] Liu Z., Shen Y., Lakshminarasimhan V.B., Liang P.P., Zadeh A.B., Morency L.P. Efficient Low-rank Multimodal Fusion With Modality-Specific Factors. Proceedings of the 56th Annual Meeting of the Association for Computational Linguistics (Volume 1: Long Papers).

[23] Tsai Y.H.H., Bai S., Liang P.P., Kolter J.Z., Morency L.P., Salakhutdinov R. Multimodal Transformer for Unaligned Multimodal Language Sequences. Proceedings of the 57th Annual Meeting of the Association for Computational Linguistics.

[24] Keller J.M., Gray M.R., Givens J.A. (1985). A fuzzy K-nearest neighbor algorithm. IEEE Trans. Syst. Man Cybern..

[25] Veličković P., Cucurull G., Casanova A., Romero A., Liò P., Bengio Y. Graph Attention Networks. Proceedings of the International Conference on Learning Representations.

[26] Brody S., Alon U., Yahav E. How Attentive are Graph Attention Networks?. Proceedings of the International Conference on Learning Representations.

[27] Guo L., Fu Y., Tan S., Wang Q., Zhang Y., Huang X., Yuan X. (2025). RadioGuide-DCN: A radiomics-guided decorrelated network for medical image classification. Bioengineering.

[28] Bresson X., Laurent T. (2017). Residual gated graph ConvNets. arXiv.

[29] Lin T.Y., Goyal P., Girshick R., He K., Dollár P. (2017). Focal Loss for Dense Object Detection. Proceedings of the 2017 IEEE International Conference on Computer Vision (ICCV).

[30] Selvamini M., Ramesh S., Sadanandan A., Chandrasekharan A., Krishnamurthi G., Murali A. (2026). Radiomics-derived classifier performance evaluation in lung nodule characterization compared with expert radiologists. Sci. Rep..

[31] Li R., Zhou L., Wang Y., Shan F., Chen X., Liu L. (2023). A graph neural network model for the diagnosis of lung adenocarcinoma based on multimodal features and an edge-generation network. Quant. Imaging Med. Surg..

[32] Saihood A.A., Hasan M.A., mahmood shnawa S., Fadhel M.A., Alzubaid L., Gupta A., Gu Y. (2024). Multiside graph neural network-based attention for local co-occurrence features fusion in lung nodule classification. Expert Syst. Appl..

[33] McWilliams A., Tammemagi M.C., Mayo J.R., Roberts H., Liu G., Soghrati K., Yasufuku K., Martel S., Laberge F., Gingras M. (2013). Probability of Cancer in Pulmonary Nodules Detected on First Screening CT. N. Engl. J. Med..

[34] Heuvelmans M.A., Walter J.E., Peters R.B., de Bock G.H., Yousaf-Khan U., van der Aalst C.M., Groen H.J.M., Nackaerts K., Ooijen P.M.V., Koning H.J. (2017). Relationship between nodule count and lung cancer probability in baseline CT lung cancer screening: The NELSON study. Lung Cancer.

